# Alternative schedules or integration strategies to maximise treatment duration with sunitinib in patients with gastrointestinal stromal tumours

**DOI:** 10.3892/ol.2014.2348

**Published:** 2014-07-11

**Authors:** MARISTELLA SAPONARA, CRISTIAN LOLLI, MARGHERITA NANNINI, VALERIO DI SCIOSCIO, CARLA SERRA, ANNA MANDRIOLI, MARIA CATERINA PALLOTTI, GUIDO BIASCO, MARIA ABBONDANZA PANTALEO

**Affiliations:** 1 Department of Specialized, Experimental and Diagnostic Medicine, S.Orsola-Malpighi Hospital, University of Bologna, Bologna 40138, Italy; 2 Interdepartmental Centre of Cancer Research ‘G. Prodi’, University of Bologna, Bologna 40138, Italy; 3 Department of Radiology, S.Orsola-Malpighi Hospital, University of Bologna, Bologna 40138, Italy; 4 Department of Digestive Disease and Internal Medicine, S.Orsola-Malpighi Hospital, University of Bologna, Bologna 40138, Italy

**Keywords:** alternative schedules, therapy management, gastrointestinal stromal tumor, sunitinib, treatment optimization, radiofrequency

## Abstract

Gastrointestinal stromal tumours (GISTs) are the most common mesenchymal tumour of the gastrointestinal tract. The advent of targeted kinase-inhibitors has revolutionised treatment strategies and clinical outcomes for patients with advanced GIST. In the majority of countries, sunitinib is the only approved second-line treatment option for advanced GIST patients, who are resistant or intolerant to imatinib. However, sunitinib is associated with various adverse events, which often result in a reduction of the dosage, and interruption or suspension of therapy. Effective therapy management is essential to obtain the maximum clinical benefit, and includes adequate side effect management as well as optimization of dosing and treatment duration. In the current study, examples of maximization of treatment with sunitinib are presented, describing three clinical cases in which therapy with sunitinib was continued via the adoption of alternative reduced schedules or an additional loco-regional treatment, in order to manage toxicities or overcome progressive disease.

## Introduction

Gastrointestinal stromal tumours (GISTs), although rare tumours overall, are the most common type of mesenchymal tumour of the GI tract. Approximately 85–90% of GISTs are associated with gain-of-function KIT gene mutations, which lead to constitutive activation of KIT kinase activity and to uncontrolled cell proliferation. A notably smaller proportion (5%) is associated with analogous mutations in PDGFRα and <10% contain no identified receptor tyrosine-kinase mutations (termed wild-type GISTs) (1–3).

Traditional cytotoxic treatments, although active in other subtypes of sarcoma, are ineffective in GISTs. Elucidating the GIST molecular pathophysiology as a mutation-driven process has enabled the development of targeted kinase-inhibitor therapies, which have revolutionised treatment strategies and clinical outcomes for patients with advanced GISTs (4,5).

Imatinib mesylate, an oral selective inhibitor of the kinase activities of KIT and PDGFRα, was the first targeted therapy to demonstrate dramatic efficacy on GISTs. Prior to imatinib, the median overall survival (mOS) of metastatic GIST patients was 19 months (6,7). However, ~4% of patients are intolerant to imatinib therapy, ~15% show primary resistance to imatinib and >80% eventually develop a secondary or acquired resistance following a median treatment time of approximately two years. Resistance most commonly develops as a result of secondary KIT mutations in clonally expanded cancer cells (8).

Sunitinib malate is the only approved second-line treatment option for advanced GIST patients who are resistant or intolerant to imatinib. Sunitinib is an oral multitargeted receptor tyrosine-kinase inhibitor (TKI) of KIT, PDGFRα, all three isoforms of the vascular endothelial growth factor receptors (VEGFR-1, -2 and -3) and various other tyrosine-kinase receptors. It consequently targets the primary kinases that are implicated in GIST pathogenesis as well as those involved in tumour-associated angiogenesis (9).

Results of a randomised, placebo-controlled, phase III study of a regimen of 50 mg/day sunitinib during an intermittent dosing schedule of 4-weeks-on treatment followed by 2-weeks-off treatment (a 4w/2w schedule) demonstrated significant efficacy and safety in patients with advanced GISTs following PD or those with an intolerance to imatinib (10). The median time to tumour progression was more than four times longer with sunitinib compared with a placebo treatment (27.3 vs. 6.4 weeks; P<0.0001) and a significant difference in OS, favouring sunitinib [hazard ratio (HR), 0.49; P=0.007] was shown.

Long-term survival data of this trial was subjected to a novel type of statistical analysis; the rank-preserving structural failure time method, which accounts for bias that is introduced by patients crossing over from a placebo to an active treatment. This analysis demonstrated the long-term OS benefit that was provided by sunitinib compared with a placebo (74.7 vs. 36.0 weeks; HR, 0.46; P<0.0001) (11). These results led to multinational approval of sunitinib in this patient population; those who have an intolerance to imatinib and/or those showing PD.

Furthermore, an open-label phase II study was conducted on a large number of patients with sunitinib administered at a lower dose on a continuous daily dosing schedule (37.5 mg/day without off-treatment periods), which demonstrated that this type of administration provided a safe and effective dosing option without additional accumulation across cycles, and no novel or unexpected adverse events (AEs) were reported (12).

However, sunitinib is associated with AEs, which are generally mild to moderate, which may lead to a dose reduction, interruption or suspension of therapy, in the long term.

The most common AEs are fatigue, diarrhoea, nausea and vomiting, skin and hair discolouration, stomatitis, hand-foot syndrome, hypertension (HTN) and hypothyroidism. At the time of data cut-off in the placebo-controlled study, treatment-associated AEs of any severity grade and of serious AEs, were reported in 83 and 20% of patients, respectively. Twenty-eight per cent of patients interrupted their treatment, 11% required a dose reduction and 9% discontinued treatment due to the AEs experienced in the study (10); similar results were reported in the next expanded access studies (13).

In addition, a pharmacokinetic/pharmacodynamic meta-analysis was performed in order to investigate the association between clinical endpoints and sunitinib exposure in patients with advanced solid tumours, including 454 patients affected by GISTs (14). This demonstrated longer time-to-progression and OS, and a trend towards a higher probability of a decrease in tumour size or halting of tumour growth in patients with the greatest exposure to sunitinib. These analyses stressed the importance of maintaining patients on a 50-mg dose, thus avoiding unscheduled dosing interruptions or ‘jerky’ consumption of sunitinib. Therefore, the effective management of AEs is crucial to obtain consistent compliance, and achieve and maintain optimal clinical efficacy (15,16).

With the aim to improve patients’ adherence and reduce sunitinib-associated side effects, numerous studies using alternative doses or schedules of sunitinib have been conducted. For example, certain patients who were affected by metastatic renal cell carcinoma (mRCC) and for whom sunitinib represented the first-line therapy, were changed from the standard 4w/2w schedule to a new 2w/1w or 7-day-on treatment followed by 3-day-off treatment schedule (7d/3d) (17,18). The results demonstrated that treatment using alternative schedules was associated with significantly decreased toxicity in patients who had initially experienced a grade 3 or greater toxicity on the 4w/2w schedule, as well as enabled a marked extension of the treatment duration.

A phase I trial in GIST patients evaluated the feasibility of administering 50 mg sunitinib for 2 weeks followed by a 1-week-off period (19). The pharmacokinetic data demonstrated that the 2w/1w schedule provided prolonged sunitinib exposure compared with the 4w/2w schedule, without significant accumulation of sunitinib between courses, and that the 2w/1w schedule was better tolerated, with only minor dose adjustments or modifications required.

In the majority of countries, sunitinib represents the only approved therapeutic option (after imatinib) for patients that are affected by GISTs. For this reason, continuing sunitinib treatment, even following progressive disease (PD), has been proposed as a promising alternative. In an open-label retrospective study, 704 patients were dichotomized based on whether sunitinib treatment was continued or terminated following PD (20). The study demonstrated that the patients who continued on sunitinib exhibited an improved clinical outcome compared with those who terminated it (mOS, 22.8 vs. 13.2 months). The results of the abovementioned study supported the adoption of this strategy in clinical practice.

Various additional inhibitors of KIT and PDGFRα kinases have been developed. However, despite promising results in imatinib/sunitinib-resistant disease control in early phase trials, to date, none but one (regorafenib, a novel, oral multikinase inhibitor) have shown benefits in prospective phase III trials. In fact, the Food and Drug Administration only recently expanded the use of regorafenib to GIST as a result of the GRID trial results (21).

In the present study, the experiences of patients affected by GIST are described, referring specifically to the management of treatment, with the aim of discussing optimization of the treatment duration and patient outcome. In particular, two clinical cases of patients who were affected by GIST and treated with alternative schedules of sunitinib are presented, in addition to the case of a patient whose therapy with sunitinib has been continued following PD using complementary loco-regional treatment, thus obtaining a prolonged clinical benefit. Finally, a hypothesis explaining these encouraging results is provided along with a comparison between our data and those of other possible treatment options that have been reported in the literature.

## Materials and methods

### Patients

Between December 2001 and June 2013, 67 patients affected by advanced GIST were treated at the Ematology and Medical Oncology Unit ‘L&A Seràgnoli’, S.Orsola-Malpighi Hospital, University of Bologna (Bologna, Italy) with sunitinib following PD or intolerance to imatinib. The primary treatment schedule adopted in our centre is 37.5 mg/day sunitinib continuously. All 67 patients were retrospectively analyzed; 64 were treated following the standard guidelines and three required personalized treatment management. In the majority of cases, sunitinib demonstrated efficacy and safety profiles similar to those reported in the previous literature (10,12,13). Patients presented with predominantly transient or self-limiting side effects, primarily managed with dose delay, temporary dose reduction or standard supportive medical treatments. However, in three cases, it was necessary to adopt tailored strategies. As the aim of the present study was to analyse alternative schedules and integrated treatment options, the present study focused mainly on these three patients. Their clinical history will be described individually as it may significantly facilitate clinical practice. All patients provided consent.

## Results

In 64 out of the 67 patients, sunitinib treatment was discontinued in favour of other standard or experimental treatment options in order to prevent severe toxicities or PD. Such options include the following: Rechallenge with imatinib, nilotinib, sorafenib, regorafenib or the best supportive care. Conversely, in three cases out of the 67, treatment with sunitinib was prolonged despite intolerance or PD through the adoption of personalized measures or treatment adjustment (Table I). The clinical management of these three patients is reported below.

### Case 1

**Figure 1:**
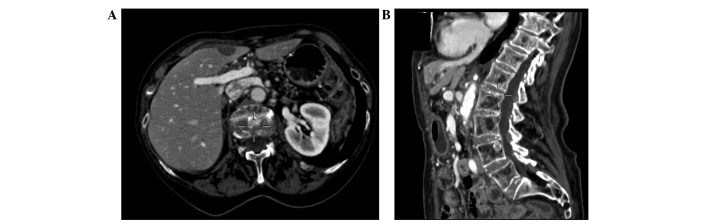
Stable liver and bone metastases during sunitinib treatment. (A) Liver metastasis in segment IV, showing hypodensity in the axial view of the computed tomography (CT)-scan. (B) Coronal view of the CT scan demonstrating multiple lytic vertebral metastases.

In October 2006, an 82-year-old female presented with an acute episode of severe anaemia. A large gastric lesion associated with multiple liver and bone metastases was detected by a computed tomography (CT)-scan. Due to the persistence of anaemia, the patient underwent a partial gastric resection and histological examination revealed a GIST. In addition, mutational analysis revealed a common KIT exon 11 mutation (p.V559D). In November 2006, the patient commenced first-line imatinib therapy at the standard dose of 400 mg/day and achieved a prolonged, stable disease. In March 2009, a new abdominal lesion of the left iliac fossa was identified. Thus, the patient was administered 800 mg/day imatinib, however, the treatment was prematurely interrupted due to severe bilateral pleural effusion. Therefore, in August 2009 the patient was enrolled in an A6181078 trial and commenced second-line therapy with sunitinib at 37.5 mg/day, which was reduced to 25 mg due to persistent leukopenia and neutropenia. Bone marrow toxicity also resulted in numerous treatment interruptions with a duration ranging from a few days up to a month. However, the disease remained stable until November 2012, when a mild dimensional increase of the known abdominal lesion was observed. However, due to the clinical benefit and the marginal focal progression, the same therapy was continued with a modified sunitinib schedule incorporating an intermittent administration (1d/1d) at a reduced dose of 25 mg in order to overcome bone marrow toxicity. In April 2013, on the final CT scan evaluation, the disease was identified to be stable (Fig. 1).

### Case 2

**Figure 2:**
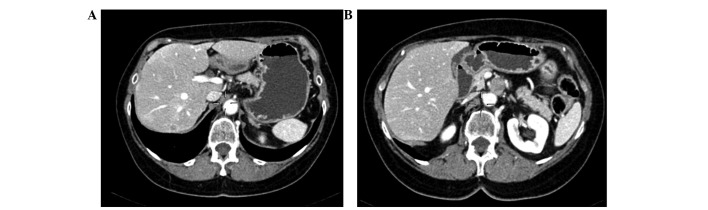
Stable liver and retro-pancreatic metastases during sunitinib treatment. (A) Liver metastasis in segment VIII, showing peripheral enhancement with a central necrotic area on computed tomography. (B) Retro-pancreatic solid lesion exhibiting stable disease with no change in size.

In November 1997, a 54-year-old female underwent surgical resection of a digiunal GIST. During the follow-up programme ~11 years later ultrasonography revealed a retro-pancreatic mass with multiple liver lesions. The diagnosis of GIST was determined by a CT-guided liver biopsy. Tumour genotyping revealed a mutation on exon 18 of the PDGFRα gene, excluding the D842V mutation. In September 2008, treatment with 400 mg/day imatinib was initiated. CT scan and contrast enhanced ultrasonography (CEUS) showed stable disease until September 2009, when CEUS demonstrated a mild increase in the size of the hepatic lesions. Thus, the dose was altered to 800 mg/day. An additional CEUS that was performed one month later indicated further mild progression. Hence, in November 2009, the patient commenced a second-line treatment of 37.5 mg/day sunitinib. Side effects caused by the treatment were characterized by diarrhoea and grade 3 HTN according to the National Cancer Institute Common Terminology Criteria for Adverse Events version 4.0 (22). Although angiotensin-converting-enzyme-inhibitors, beta-blockers, calcium-antagonists and diuretics were administered, the patient’s blood pressure remained particularly difficult to control. Thus, the sunitinib schedule was modified to 25 mg/day continuously, which resulted in the improved control of AEs. Any attempt to reintroduce the standard dose of 37.5 mg/day was characterized by a recurrence of diarrhoea and blood pressure instability. Therefore, since June 2010, the patient has continuously been treated with an alternative schedule of sunitinib; the CT scans in Fig. 2 demonstrate the stability of the disease.

### Case 3

**Figure 3:**
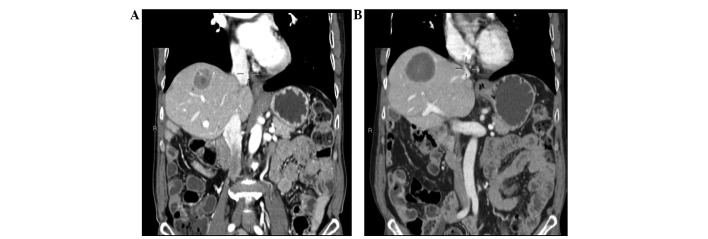
Liver metastasis treated with sunitinib and integrated with treatment using radiofrequency ablation/percutaneous ethanol injection (RFA/PEI). (A) Coronal view of the computed tomography (CT)-scan, following 50 months of sunitinib therapy and 24 months post-surgery, demonstrates a new single liver metastasis in segment VIII. (B) CT scan following treatment with RFA/PEI demonstrating complete destruction of the resistant nodule.

In December 2003, a 58-year-old male underwent surgical resection for a GIST in the ileum. The mutational analysis showed a deletion at exon 11 of the KIT gene (p.V569_Y578 del.). In January 2005, during follow-up, a CT scan detected three liver metastases. Treatment with 400 mg imatinib was initiated, however, the imatinib was administered irregularly as it caused deep fatigue, mucositis and a skin-rash. In July 2005, the administration of imatinib was permanently discontinued. Due to a lack of novel approved drugs, the patient underwent three wedge resections of the liver, however, in January 2006, a CT scan revealed six new liver lesions. In July 2006, the patient was referred to the Ematology and Medical Oncology Unit ‘L&A Seràgnoli’, S.Orsola-Malpighi Hospital, University of Bologna and was enrolled in the A6181036 protocol; commencing sunitinib, initially at a daily dose of 50 mg (4w/2w) and subsequently at the continuative dose of 37.5 mg/day. The CT scan revealed a clear response to therapy, with a reduction in size of the primary lesion and a decrease in tumour density of the remaining lesions. In September 2008, due to the long-term stability of the disease, the patient underwent a third surgical intervention (multiple wedge resections) and surgery was considered to be complete. Immunohistochemistry determined the diagnosis of a GIST, which was characterized by a good histological response to therapy. Treatment with sunitinib was continued following surgery and periodic CT scans were performed until September 2010, when a liver relapse of 37 mm was detected in segment VIII. Since it was the only site of relapse and there was no alternative approved therapy at that time, a loco-regional treatment approach with radiofrequency (RFA) and/or percutaneous ethanol injection (PEI) was adopted. Thus, in December 2010, considering the site and size of the lesion, an RFA + PEI treatment was performed and the post-procedure CT scan revealed a necrotic area without any sign of active disease (Fig. 3). This result has been maintained over time and the sunitinib treatment has been continued.

## Discussion

Sunitinib is the only approved second-line treatment option for patients with advanced GIST, who are resistant or intolerant to imatinib. It provides clinical benefit following the failure of imatinib treatment and it has been shown to be more active than first-line treatments in patients with wild-type GIST and KIT exon 9 mutations (all of which are relatively resistant to imatinib), as well as those with KIT exon 11 mutations. Sunitinib-associated activity was also observed in patients with secondary KIT mutations in exons 13 and 14 (23).

Sunitinib-associated AEs are generally mild to moderate, however, in clinical practice, intolerance caused by toxicities frequently leads to dose reductions and/or breaks in treatment. Therefore, effective therapy management is key to avoid inadequate dosages and loss of treatment efficacy.

With the advent of regorafenib, a newly approved therapeutic agent for GISTs, the likelihood of treating this type of tumour is improved. However, regorafenib is currently not globally available and it is associated with considerable side effects. In the GRID trial, 98% of patients presented treatment-associated AEs of all grades and 61% presented serious AEs, including hand-foot syndrome, skin reactions, diarrhoea, abdominal pain and fever (21).

Hence, in current clinical practice, the most common therapeutic alternatives upon sunitinib failure are as follows: Rechallenge of imatinib; nilotinib; sorafenib; or the best supportive care (24). Overall, the clinical benefit rate (CBR), median progression free survival (mPFS) and mOS that were obtained with those treatment options, ranged between 11 and 42%, from 2.1 to 4.9 months, and from 2.4 to 11.8 months, respectively. These results, together with those obtained with regorafenib, are summarized in Table II.

Three clinical cases have been presented in which therapy, using sunitinib, was continued via the adoption of alternative reduced schedules or an additional loco-regional treatment, in order to manage toxicities or overcome PD. These decisions were predominantly driven by the absence of alternative approved therapeutic agents at the time of progression or due to an intolerance to sunitinib. Furthermore, the selection of alternative treatments was reinforced by the long response that was previously obtained from administering sunitinib to these patients.

In case 1, an elderly women affected by a metastatic GIST at diagnosis was treated with sunitinib for 46 months in total. Following the first cycle, a dose reduction, initially to 25 mg/day, then to 25 mg/day 1d/1d was prescribed (due to grade 3 hematologic AEs) and improved the bone marrow toxicity profile, whilst maintaining disease control until the present.

Although evaluation of sunitinib blood levels was not conducted in the patients in the current study, it is likel that adequate sunitinib levels to prevent PD have been maintained, despite does reduction, due to: i) Old age (88-years-old at the time of switching to an alternative schedule) with consequent age-associated decline in sunitinib metabolism, and changes in the therapeutic agent and the availability of metabolites (25); and ii) low body mass index (BMI) of 17.98.

Concerning BMI, certain available results demonstrate the importance of adapting the dosage of cytotoxic chemotherapy to weight, or derivatives of weight, such as BMI, whilst a small quantity of data are available regarding BMI and targeted therapies (26,27). Thus, sunitinib is applied at a constant dose of 50 mg/day, 4w/2w or 37.5 mg/day continuously, not accounting for several sources of interindividual variance, such as body size, which are often considerable, explaining differences in therapeutic agent concentration, metabolism and ultimately tolerance. This is an interesting and novel area of research, however, further studies are required to obtain definitive results.

In the next case reported, a 70-year-old female showed rapid PD during imatinib treatment (13 months). Conversely, the patient obtained good disease control when administered with sunitinib. However, due to diarrhoea and HTN, the patient required treatment adjustment to 25 mg/day. Again, the patient’s tolerance improved and disease control was preserved.

The clinical history of this patient during sunitinib treatment was marked by severe HTN. This AE is characterised in the most recent literature as a significant biomarker of sunitinib efficacy (28,29). In addition to hypothyroidism and hand-foot syndrome, it is defined as a mechanism-based toxicity as it is caused by the mechanism of action of sunitinib (30,31).

HTN affects 11–28% of GIST patients and generally begins at the end of the first or second treatment cycle (10,12). Hypotheses have been proposed concerning the occurrence of this event, according to which the administration of sunitinib may lead to an increase in vascular resistance by reducing the production of nitric oxide. Furthermore, inhibition of VEGFR by sunitinib may result in a density decrease of the small arterioles and capillaries (vascular rarefaction) (32).

HTN has recently been shown to correlate with the clinical outcome in mRCC (28). Subsequently, a retrospective analysis of three phase I–III trials (319 patients) was conducted to examine the correlations between sunitinib-associated HTN and antitumour efficacy in GIST (29). The results of the study showed that HTN correlates with an improved overall response rate [16% in patients with HTN vs. 3% in patients without HTN (P=0.004)], PFS [34 weeks in patients with HTN vs. 16 weeks in patients without HTN (P<0.0001)] and OS [87 weeks in patients with HTN vs. 53 weeks in patients with no HTN (P=0.0003)].

In the last case, a 67-year-old male who benefited from sunitinib for a particularly long duration (50 months) was described; complete liver surgery was planned 26 months following the initiation of sunitinib. No relevant AE arose other than moderate fatigue. Thus, with the onset of a single liver metastasis, a current treatment was integrated using a loco-regional approach, rather than considering a rechallenge of imatinib or searching for novel experimental therapeutic agents.

Liver surgery combined with systemic therapy is an established technique to improve the outcome of patients affected by metastases from multiple tumours. The same approach has been investigated in GISTs using surgery/TKI therapy integration, which showed favourable results, especially in imatinib-responder patients (33,34). A small number of cases of sunitinib-responders have also been reported (35).

RFA appears to be an interesting option for the treatment of small-size liver GIST metastases. It has shown encouraging results in primary and metastatic liver tumours measuring ≤3 cm, obtaining a rate of local control that is equivalent to that of surgery resection, with reduced morbidity and mortality rates. A retrospective study was conducted to assess the role of RFA in the multimodality management of GIST, demonstrating that RFA is a feasible, safe and useful option in patients with liver metastasis of GIST (36). This is the case, particularly when performed upon achievement of the optimum clinical response to TKIs and in combination with post-RFA resumption of the therapeutic agent. In the present case, RFA was used against local progression under sunitinib therapy, with the aim of ablating individual lesions, which developed a resistance to sunitinib prior to spreading, thus allowing the continuation and prolonging the efficacy of the second-line systemic therapy. As a result of this, the patient was able to continue sunitinib to date, maintaining disease control for an additional 30 months following thermoablation.

In conclusion, sunitinib represents an effective therapeutic treatment against GISTs, exhibiting a direct antitumour and antiangiogenic effect. This is particularly true in a subgroup of patients whose boundaries have not yet been precisely identified. However, sunitinib treatment is characterized by multiple, varying AEs. The present report of clinical cases and the observations regarding dose adjustment and the dose/efficacy correlation may facilitate the management of patients who are affected by GISTs. Notably, the patients in the current report are representative of the general population receiving sunitinib.

The decision to continue administering sunitinib (despite PD) by using alternative schedules to overcome AEs was due to the following: i) A lack of approved third-line therapies; and ii) the poor likelihood of restoring the efficacy of an imatinib rechallenge considering the patient’s mutational status, and previous response and tolerability to first-line therapies.

These observations remain relevant, when the increasing knowledge on this rare type of tumour drives the development and evaluation of TKIs. Furthermore, physicians may consider an increasingly wide spectrum of treatment options, basing their decision on the specific characteristics and clinical history of each patient, with the aim of maximizing the duration of each therapeutic method and, ultimately, the overall sequential treatment strategy.

## Figures and Tables

**Table I:** Characteristics of three patients who continued sunitinib despite disease progression or intolerance, through adoption of an AS or an an IS.

				Duration of therapy, months (regimen)			
				
Case	Age^a^, years	Gender	Risk category at diagnosis	Imatinib: 400 mg/day	Imatinib: 800 mg/day	Sunitinib: SS	Sunitinib: AS or IS
1	85	F	Metastatic	28	5	7	32 (25 mg/day) 7 (25 mg 1d/1d^b^)
2	66	F	Low	12	1	7	36 (25 mg/day)
3	61	M	Moderate	6	0	53	30 (post RFA)

a On initiation of sunitinib treatment;

b 1-day-on treatment, 1-day-off treatment.

F, female; M, male; SS, standard schedule; AS, alternative schedule; IS, integration strategy; RFA, radiofrequency ablation.

**Table II:** Patterns of the most common third-line therapies and associated CBRs, mPFS and mOS.

Treatment type, no. of patients (ref)	CBR (%)	mPFS (months)	95% CI	mOS (months)	95% CI
Rechallenge with imatinib, 40 (23)	25	2.9	2.2–3.5	7.5	4.0–10.9
Nilotinib, 67 (23)	35	4.1	2.8–5.3	11.8	7.2–16.3
Sorafenib, 55 (23)	42	4.9	2.2–7.6	10.7	7.1–14.2
Regorafenib, 133 (21)	53	4.8	1.4–9.2	-	-
Best supportive care, 18 (23)	11	2.1	1.3–2.8	2.4	1.8–2.9

CBR, clinical benefit rate; mPFS, median progression free survival; CI, confidence interval; mOS, median overall survival.
